# Analysis of force and displacement of anchor systems under the non-limit active state

**DOI:** 10.1038/s41598-021-04668-9

**Published:** 2022-01-25

**Authors:** Guang-Wen Fang, Yan-Peng Zhu, Shuai-Hua Ye, An-Ping Huang

**Affiliations:** 1 School of Civil Engineering, Lanzhou University of Technology, Lanzhou, 730050 China; 2 Western Engineering Research Center of Disaster Mitigation in Civil Engineering of Ministry of Education, Lanzhou University of Technology, Lanzhou, 730050 China

**Keywords:** Engineering, Civil engineering

## Abstract

This paper proposes a concept of soil shear strength exertion around an anchorage section to analyze and calculate the anchor’s force and displacement. First, based on the Mohr stress circle, the numerical relationship between the value of the internal friction angle exertion, the value of the cohesion exertion, and the displacement are established under the non-limit active state. According to the interaction mechanism between the anchorage section and the surrounding soil, this paper obtains the corresponding relationship between the value of the soil’s shear strength exertion and displacement under the non-limit active state. Then, according to the load transfer principle of the anchorage section, the basic differential equation is established. The differential equation of the relative displacement distribution along the length of the anchorage section under the non-limit active state is further derived. And the calculation and analysis formula of the shear stress and relative displacement in the anchorage section considering the process of the soil shear strength exertion is also obtained. Finally, this paper compares the calculation method with the hyperbolic model calculation method for the same example. The results verify the accuracy of the calculation method.

## Introduction

In a slope’s supporting structure reinforced by an anchor, the main force-receiving and force-transmitting components are the anchors; that is, the lateral pressure generated by the weight of the slope soil and the external load will transfer to the free section through the connection between the anchor and the supporting structure. The lateral pressure will then transfer from the free section to the anchorage section in the stable soil layer. This results in a relative displacement trend between the anchorage section and the surrounding soil. The soil around the anchorage section also produces side friction that prevents displacement of the anchorage section. This achieves equilibrium and ensures the safety and stability of the slope. As the main force-receiving and force-transmitting component of this kind of support system, the anchor’s deformation (displacement) directly affects the overall reinforcement effect of the slope support system. Several studies have investigated the anchor’s force, displacement, and displacement composition to determine the relationship between force and displacement under normal working conditions.

In the 1970s, Fujita et al.^1^ proposed a load–displacement calculation method, which assumed that the shear stress on the surface of the anchor presents an elastic–plastic relationship with its relative displacement. This explained why, with an increasing load, the axial force of the anchor increases gradually from the top to the bottom of the anchorage section. However, this method does not consider the non-linear relationship between the shear stress and the relative displacement of the anchorage section; it considers only that shear stress increases linearly to the limit value and remains unchanged in the later period. By monitoring the anchor in cohesive soil, Evangelista and Sapio^2^ concluded that the side friction resistance of the anchorage section was distributed non-uniformly along its length. Since then, other studies^3–6^ have investigated-theoretically, experimentally, and numerically-the non-uniform distribution law of the side friction resistance and the relative displacement of the anchor, together with the anchor’s load transfer mechanism. Stress influencing factors, effective length, and safety reserve of the anchorage section have also been examined. To study the axial force change, load transfer mechanism, creep behavior, and the anchor’s loading and unloading performance, Benmokrane et al.^7^ suggested a monitoring method using vibrating wire pressure gauge and verified it by experiments, which provided a technical means for further studies of the anchor load transfer mechanism. After assuming that the side friction resistance and the relative displacement increase linearly, Zhang and Tang^8^ analyzed the displacement composition and established a hyperbolic function model of anchor load transfer. And from the analytical solution, the distribution law and influencing factors of shear stress and the relative displacement along the length of the anchorage section are obtained. Kim^9^ used a simple beam-spring numerical model to study the load transfer mechanism of the anchor based on the anchor’s measured data. Using ABAQUS finite element software to simulate the load transfer mechanism of the anchor and comparing it to the field measured data, Kim et al.^10^ verified the feasibility of the numerical simulation model. Xiao and Chen^11^ analyzed the mechanical characteristics of the anchorage section based on the actual non-linear distribution of shear stress and load transfer mechanism. According to the softening characteristics of the anchorage section, the relationship between the shear stress and the relative displacement was simplified as a three-folding-lines model, and the variation of the stress and displacement of the anchorage section with the load on the anchor was discussed. Wei et al.^12^ used the hyperbolic load transfer function to analyze the load and displacement of tension anchor according to the mechanism of the side friction resistance of the anchorage section and the relative displacement between the anchor and the soil. Ren et al.^13^ proposed an analytical method for calculating the mechanical behavior of the anchors in the entire process based on the tri-linear bond-slip model. This method calculated the change in the anchor’s load displacement, interface shear stress distribution, and the axial force. Ke et al.^14^ conjectured that when the shear stress between the anchorage section and surrounding soil is less than the soil’s shear strength, the interface of the anchorage section is in an elastic state and meets the deformation coordination conditions. Based on their hypothesis, this paper proposes a relative displacement calculation model for analyzing the anchorage’s load transfer mechanism to establish the calculation formulas for axial force, shear stress, and displacement around the anchorage section along its length. Xu et al.^15^ used a high-pressure jet mixing anchor pile as the research object and analyzed the load and displacement transfer law of this type of anchor under an external load by using a hyperbolic calculation model. Zhang and Chen^16^ divided the shear stress distribution into three stages—elastic, plastic, and failure—according to the mechanical mechanism. Given the uncertainty of the location of the shear stress peak in the working state of the anchor, the authors elaborated on the reasons for the shear stress peak in each stage. The present paper introduces the transfer coefficient and derives an analytical formula for load transfer that reflects the three-stage change law. Xu and Yin^17^ used Brillouin Optic Time Domain Analysis sensor technology to monitor the whole process of slope construction of the Glass Fibre Reinforced Polymer anchor and obtained the shear stress distribution form of the anchor, which provides a new method to measure anchor related parameters. Ni et al.^18^ derived a generalized non-linear softening load transfer model for the load-settlement response of a single pile under axial load, which also provides a new way to analyze the load transfer mechanism of the anchor. Liu et al.^19^ established a method for evaluating the bearing capacity of the anchor based on the pull-out test and theoretical basis of the anchors in rocks. This method was used to analyze the interface properties of the anchorage section. To study the anchor’s load transfer mechanism in the pulling experiment, Fabris et al.^20^ precisely monitored the force and deformation of the entire anchor by installing an optical fiber sensor along the steel bar. The authors obtained the anchor’s load–displacement curve and crack development law in the pull-out test. Bryson and Giraldo^21^ described the variation of side friction resistance and relative displacement of the anchorage section using different strain-softening load transfer functions and proposed a load–displacement calculation method based on the t-z curves. The calculation method shows that the side friction resistance of the anchorage section has a significant influence on the anchor’s load displacement. Wang et al.^22^ established the calculation model of a grouted anchor considering the coupling effect of tension and torsion in the anchorage section and studied the force transfer characteristics of a grouted anchor under rotating or non-rotating conditions and the asymptotic failure mechanism of the anchorage section.

Based on the above research and analysis, calculations for the anchor’s force and displacement can be divided into two parts: the free section and the anchorage section. Specific calculations and analysis of these are summarized as follows:

1. The deformation calculation of the free section is based on the theory of elastic bodies; that is, the deformation of the free section is obtained by the force of the anchor and the stiffness of the free section.
2. For calculating the relative displacement of the anchorage section, most studies obtained the distribution form of side friction resistance (shear stress) and relative displacement along the length of anchor assuming that the distribution relationship between the side friction resistance and the relative displacement of the anchorage section is non-linear (according to the variation trend of the measured data), and deduced the calculation formula of the anchor’s relative displacement.

According to the analysis of the above two points, the calculation method for the free section is reasonable. However, the calculation method of the anchorage section does not analyze the variation law of side friction resistance with relative displacement at the theoretical level, only assuming a variation relationship based on the variation trend of the measured data. Therefore, it is necessary to deduce the non-linear change law of the relative displacement and shear stress of the anchorage section and the corresponding calculation formula at a theoretical level.

Using the analysis of the formation process of the side friction resistance of the anchorage section, the present paper considers the side friction resistance between the anchorage section and the surrounding soil to be equivalent to the value of shear strength exertion of the surrounding soil; that is, the shear stress between the anchorage section and the surrounding soil after the relative displacement of the anchorage section. Therefore, to determine the relationship between the side friction resistance and relative displacement, we consider only the relationship between the value of shear strength exertion and relative displacement. Indeed, the value of shear strength exertion is closely related to the value of internal friction angle exertion and cohesion exertion. The internal friction angle and cohesion of the surrounding soil will gradually increase from the initial value to the limit value with an increase in the relative displacement of the anchorage section. The corresponding value of shear strength exertion around the anchorage section will also gradually increase from the initial value to the limit value until it reaches the shear strength’s limit value and the anchorage section is damaged. It should be noted that, according to the actual working state and failure form of the slope reinforced by the anchor, the process of shear strength exertion examined in this paper refers specifically to the change from the static state to the limit active state (non-limit active state); that is, the process of the anchor pulling out after being stressed. Therefore, the change analyzed below refers to the non-limit active state.

Based on the above analysis, in order to calculate the deformation of the free section under the elastic theory, given the limitations in the calculation and analysis of the anchor’s force and displacement, this paper introduces the concept of the process of soil shear strength exertion around the anchorage section (specifically, the change of the value of soil shear strength exertion with the relative displacement of the anchorage section), which is used to analyze and calculate the non-linear change law of the relative displacement of the anchorage section and the value of soil shear strength exertion. To obtain a more accurate calculation formula of the anchor force and displacement, the non-linear function analysis model of the anchor load transfer along the anchorage section is established under the non-limit active state.

## The relationship between the value of the shear strength parameters exertion and displacement under the non-limit active state

For cohesive soils, the shear strength parameters include the internal friction angle and cohesion, of which exertion process are directly related to the displacement of the soil. According to the relationship between the force and displacement of the anchorage section, this paper considers that the side friction resistance between the anchorage section and the surrounding soil gradually increases from the initial value to the extreme value with an increase in the relative displacement between the anchorage section and the surrounding soil. This eventually leads to the anchor pulling out (when the strength of the steel bar of the anchor is sufficient). At the same time, the side friction resistance between the anchorage section and soil can be theoretically equivalent to the value of soil shear strength exertion, so the former can also be expressed by the internal friction angle and cohesion at the anchorage section.

### Relationship between the value of internal friction angle exertion and displacement under the non-limit active state

**Figure 1:**
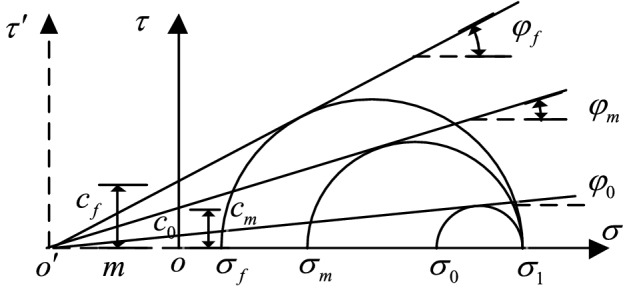
Mohr stress circle.

The Mohr stress circle of cohesive soil is shown in Fig. 1. If the ordinate is translated as $m$ so that the three curves intersect the abscissa at point $o^{\prime}$, the meaning of the parameters in the Fig. 1 is as follows:

$\sigma_{1}$ Maximum principal stress under initial state.

$\sigma_{0}$ Minimum principal stress under initial state.

$\sigma_{m}$ Minimum principal stress under non-limit active state.

$\sigma_{f}$ Minimum principal stress under limit active state.

$\varphi_{0}$ Internal friction angle under initial state.

$\varphi_{m}$ Internal friction angle under non-limit active state.

$\varphi_{f}$ Internal friction angle under limit active state.

$c_{0}$ Cohesion under initial state.

$c_{m}$ Cohesion under non-limit active state.

$c_{f}$ Cohesion under limit active state.

According to the actual force and damage of the anchorage section, we consider that $\sigma_{1}$ keeps the vertical direction unchanged, while $\sigma_{0}$, $\sigma_{m}$, and $\sigma_{f}$ are the horizontal direction. Therefore, from Fig. 1 and the characteristics of the Mohr stress circle, the following relationship can be obtained^23^.

1 $$\sin \varphi_{f} = \frac{{\left( {\sigma_{1} - \sigma_{f} } \right)/2}}{{\left( {\sigma_{1} + \sigma_{f} } \right)/2 + m}} = \frac{{\left( {\sigma_{1} + m} \right) - \left( {\sigma_{f} + m} \right)}}{{\left( {\sigma_{1} + m} \right) + \left( {\sigma_{f} + m} \right)}}$$

2 $$\sin \varphi_{m} = \frac{{\left( {\sigma_{1} - \sigma_{m} } \right)/2}}{{\left( {\sigma_{1} + \sigma_{m} } \right)/2 + m}} = \frac{{\left( {\sigma_{1} + m} \right) - \left( {\sigma_{m} + m} \right)}}{{\left( {\sigma_{1} + m} \right) + \left( {\sigma_{m} + m} \right)}}$$

When $\sigma_{1}^{\prime } = \sigma_{1} + m$, $\sigma_{0}^{\prime } = \sigma_{0} + m$, $\sigma_{m}^{\prime } = \sigma_{m} + m$, $\sigma_{f}^{\prime } = \sigma_{f} + m$, Eqs. (1) and (2) can be changed as follows:

3 $$\sin \varphi_{f} = \frac{{\sigma_{1}^{\prime } - \sigma_{f}^{\prime } }}{{\sigma_{1}^{\prime } + \sigma_{f}^{\prime } }} = \frac{{\left( {\sigma_{1}^{\prime } - \sigma_{0}^{\prime } } \right) + \left( {\sigma_{0}^{\prime } - \sigma_{f}^{\prime } } \right)}}{{\left( {\sigma_{1}^{\prime } + \sigma_{0}^{\prime } } \right) - \left( {\sigma_{0}^{\prime } - \sigma_{f}^{\prime } } \right)}}$$

4 $$\sin \varphi_{m} = \frac{{\sigma_{1}^{\prime } - \sigma_{m}^{\prime } }}{{\sigma_{1}^{\prime } + \sigma_{m}^{\prime } }} = \frac{{\left( {\sigma_{1}^{\prime } - \sigma_{0}^{\prime } } \right) + \left( {\sigma_{0}^{\prime } - \sigma_{m}^{\prime } } \right)}}{{\left( {\sigma_{1}^{\prime } + \sigma_{0}^{\prime } } \right) - \left( {\sigma_{0}^{\prime } - \sigma_{m}^{\prime } } \right)}}$$

According to the hyperbolic relationship between radial stress and radial strain:

5 $$\sigma_{0}^{\prime } - \sigma_{m}^{\prime } = \frac{{\varepsilon_{m} }}{{a + b\varepsilon_{m} }}$$

where $\varepsilon_{m}$ is the radial strain under the non-limit active state, $a$ and $b$ are the test parameter related to the soil properties.

When the soil around the anchorage section is under the non-limit active state, Eq. (5) can be expressed as follows:

6 $$\sigma_{0}^{\prime } - \sigma_{f}^{\prime } = \frac{{\varepsilon_{f} }}{{a + b\varepsilon_{f} }}$$

When $\varepsilon_{m} \to \infty$, Eq. (5) can be expressed as follows:

7 $$b = \left. {\frac{1}{{\sigma_{0}^{^{\prime}} - \sigma_{m}^{^{\prime}} }}} \right|_{{\varepsilon_{m} \to \infty }} = \frac{1}{{\left( {\sigma_{0}^{^{\prime}} - \sigma_{m}^{^{\prime}} } \right)_{u} }}$$

where $\left( {\sigma_{0}^{\prime } - \sigma_{m}^{\prime } } \right)_{u}$ is the asymptotic value of $\sigma_{0}^{\prime } - \sigma_{m}^{\prime }$ when $\varepsilon_{m} \to \infty$.

This paper introduces the damage ratio $R_{f}$ to express the severity of soil damage around the anchorage section, so the damage ratio $R_{f}$ can be expressed as follows:

8 $$R_{f} = \frac{{\left( {\sigma_{0}^{\prime } - \sigma_{m}^{\prime } } \right)_{f} }}{{\left( {\sigma_{0}^{\prime } - \sigma_{m}^{\prime } } \right)_{u} }} = b\left( {\sigma_{0}^{\prime } - \sigma_{f}^{\prime } } \right)$$

where $\left( {\sigma_{0}^{\prime } - \sigma_{m}^{\prime } } \right)_{f}$ is the value of $\sigma_{0}^{\prime } - \sigma_{m}^{\prime }$ when it is destroyed; that is, the value of $\sigma_{m}^{\prime } { = }\sigma_{f}^{\prime }$; the value of $R_{f}$ is generally between $0.75\;{ - }\;1.0$.

According to Eqs. (6) and (8), $\varepsilon_{f}$ can be expressed as follows:

9 $$\varepsilon_{f} = \frac{{aR_{f} }}{{b\left( {1 - R_{f} } \right)}}$$

$\eta$ is defined as the proportion of the strain of the soil element around the anchorage section in the total allowable strain; that is, $\eta = \varepsilon_{m} /\varepsilon_{f}$, then:

10 $$\varepsilon_{m} = \eta \varepsilon_{f}$$

Under initial state:

11 $$\sigma_{0}^{\prime } = K_{0} \sigma_{1}^{\prime }$$

where $K_{0}$ is the coefficient of static earth pressure.

According to Eqs. (3), (8), and (11), $b$ can be expressed as follows:

12 $$b = \frac{{1 + \sin \varphi_{f} }}{{\left( {1 + K_{0} } \right)\sin \varphi_{f} \sigma_{1}^{\prime } - \left( {1 - K_{0} } \right)\sigma_{1}^{\prime } }}$$

According to Eqs. (4), (5), and (10)-(12), $\sin \varphi_{m}$ and $\varphi_{m}$ can be expressed as follows:

13 $$\sin \varphi_{m} = \frac{{\left( {1 - R_{f} + \eta R_{f} } \right)\left( {1 - K_{0} } \right)\left( {1 + \sin \varphi_{f} } \right) + \eta \sin \varphi_{f} \left( {1 + K_{0} } \right) - \eta \left( {1 - K_{0} } \right)}}{{\left( {1 - R_{f} + \eta R_{f} } \right)\left( {1 + K_{0} } \right)\left( {1 + \sin \varphi_{f} } \right) - \eta \sin \varphi_{f} \left( {1 + K_{0} } \right) + \eta \left( {1 - K_{0} } \right)}}$$

14 $$\varphi_{m} = \arcsin \left[ {\frac{{\left( {1 - R_{f} + \eta R_{f} } \right)\left( {1 - K_{0} } \right)\left( {1 + \sin \varphi_{f} } \right) + \eta \sin \varphi_{f} \left( {1 + K_{0} } \right) - \eta \left( {1 - K_{0} } \right)}}{{\left( {1 - R_{f} + \eta R_{f} } \right)\left( {1 + K_{0} } \right)\left( {1 + \sin \varphi_{f} } \right) - \eta \sin \varphi_{f} \left( {1 + K_{0} } \right) + \eta \left( {1 - K_{0} } \right)}}} \right]$$

For normally consolidated soil, when $\eta = 0$, the initial value of internal friction angle can be obtained from Eq. (14):

15 $$\varphi_{0} = \arcsin \left( {\frac{{1 - K_{0} }}{{1 + K_{0} }}} \right)$$

where, for cohesive soil, there is $K_{0} = 0.95 - \sin \varphi_{f}$, where $\varphi_{f}$ is the limit value of internal friction angle of soil measured by the conventional geotechnical test.

When $\eta = 1$, the soil around the anchorage section is in the ultimate active state, and the internal friction angle is fully exerted and reaches the limit value. According to Eq. (14), $\varphi_{m} = \varphi_{f}$.

In summary, Eq. (14) expresses the non-linear relationship between the internal friction angle of the cohesive soil and the displacement of the soil around the anchorage section under the non-limit active state. From Eq. (14), we can conclude that the exertion degree of the internal friction angle is related only to the initial earth pressure coefficient $K_{0}$, the damage ratio $R_{f}$ (usually between $0.75\;{ - }\;1.0$), the displacement specific gravity $\eta$, and the limit value of internal friction angle $\varphi_{f}$.

### Relationship between the value of cohesion exertion and displacement under the non-limit active state

During soil deformation, the process of shear strength exertion is more complex, especially for cohesive soil: the cohesion affects the degree of soil shear strength exertion. In the shear strength formula based on the Mohr–Coulomb criterion, cohesion represents the shear strength of the soil when the normal stress of failure surface is zero, so its exertion degree also changes from its initial value to limit value with the change of displacement; that is, cohesion $c_{m}$ changes non-linearly between $c_{0} \;{ - }\;c_{f}$. Therefore, it can be obtained from the geometric relationship in Fig. 1:

16 $$c_{f} \cot \varphi_{f} = c_{m} \cot \varphi_{m}$$

By adjusting the sequence of Eq. (16), the relationship between cohesion and displacement under the non-limit active state can be expressed as follows:

17 $$c_{m} = \frac{{\tan \varphi {}_{m}}}{{\tan \varphi_{f} }}c_{f}$$

where $\varphi_{m}$ is the internal friction angle under the non-limit active state, which can be obtained from Eq. (14); $\varphi_{f}$ and $c_{f}$ represent the limit values of internal friction angle and cohesion respectively, both of which are obtained from conventional geotechnical tests.

From Eqs. (14) and (17), we can conclude that the exertion degree of cohesion is related only to the initial earth pressure coefficient $K_{0}$, the damage ratio $R_{f}$ (usually between 0.75 and 1.0), the displacement specific gravity $\eta$, the limit value of internal friction angle $\varphi_{f}$, and the limit value of cohesion $c_{f}$.

## Analysis of anchor force and displacement under the non-limit active state

**Figure 2:**
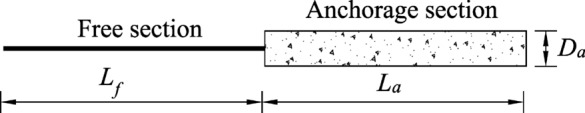
The structure of anchor.

According to the force characteristics of the anchorage, its displacement $S^{a}$ consists of the elastic deformation of the steel bar in the free section $S_{fe}$, the tensile deformation of the anchorage section $S_{ae}$, the relative displacement between the anchorage section and the surrounding soil $S_{as}$, the plastic deformation of the free section and the anchorage section, the straightening of the steel bar, and the contact gap between the anchorage and the base plate. Because the plastic deformation of the anchor is generally small and can be ignored, and the contact gap in the anchor displacement eliminated by pretensioning, the anchor displacement can be expressed as follows (the structure of anchor is shown in Fig. 2):

18 $$S^{a} = S_{fe} + S_{ae} + S_{as}$$

### Analysis of force and displacement of the free section under the non-limit active state

The anchor’s body is usually a steel bar or steel strand. When the force is below the limit value, the free section has elastic deformation. In the supporting form of reinforcing the slope with a bolt, the free section is mainly affected by external tension. Therefore, according to Hooke's law, it can be concluded that:

19 $$S_{fe} = \frac{{PL_{f} }}{{E_{s} A_{s} }}$$

where $P$ is the external tension of the anchor; $L_{f}$ is the length of free section; $E_{s}$ is the elastic modulus of free section; $A_{s}$ is the cross-sectional area of free section.

### Analysis of force and displacement of the anchorage section under the non-limit active state

**Figure 3:**
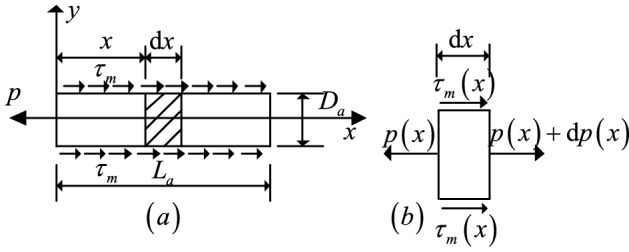
Load transfer analysis of the anchorage section.

To analyze the force and displacement of the anchorage section, this paper adopts the load transfer theory and takes the anchorage section as the isolation unit. Its force is shown in Fig. 3a. Figure 3b shows the force analysis of any anchorage section element.

#### Establishing the basic differential equation of the anchorage section under the non-limit active state

According to the displacement of the anchorage section, the total displacement of this section $S_{a}$ consists of its tensile deformation $S_{ae}$ and the relative displacement between the anchorage section and the surrounding soil $S_{as}$; that is,

20 $$S_{a} = S_{ae} + S_{as}$$

According to the displacement variation characteristics of the anchorage section and the free section, the total displacement of the anchorage section $S_{a}$ is equal to the relative displacement between the top of the anchorage section and the surrounding soil $S_{as}$ at the junction position; that is,

21 $$S_{a} = S_{as} \left| {_{x = 0} } \right.$$

By analyzing the axial static equilibrium condition of any element in the anchorage section in Fig. 3b, it can be concluded that:

22 $$\frac{{{\text{d}}p\left( x \right)}}{{{\text{d}}x}} = \pi D_{a} \tau_{m} \left( x \right)$$

where $p\left( x \right)$ is the axial tension on the unit of anchorage section; $\tau_{m} \left( x \right)$ is the shear stress on the surface of the unit of the anchorage section, and its essence is the value of shear strength exertion between the unit of anchorage section and the surrounding soil; $D_{a}$ is the diameter of the anchorage section.

According to Hooke’s law, the relationship between the axial force of the anchorage section and the tensile deformation of the anchorage section is as follows:

23 $$p\left( x \right) = \frac{{\pi D_{a}^{2} E_{a} }}{4} \cdot \frac{{{\text{d}}S_{ae} \left( x \right)}}{{{\text{d}}x}}$$

where $E_{a}$ is the effective elastic modulus of the anchorage section, and its formula is as follows:

24 $$E_{a} = \frac{{E_{s} A_{s} + E_{g} A_{g} }}{{A_{s} + A_{g} }}$$

where $E_{s}$ is the elastic modulus of the anchor’s steel bar; $E_{g}$ is the elastic modulus of the slurry of the anchorage section; $A_{s}$ is the cross-sectional area of the anchor’s steel bar; $A_{g}$ is the cross-sectional area of the slurry of the anchorage section.

According to the load transfer theory and Eqs. (20) to (23), the governing differential equation of the anchorage section is as follows (the detailed derivation of Eq. (25) is shown in Appendix A.):

25 $$\frac{{d^{2} S_{as} \left( x \right)}}{{dx^{2} }} - \frac{4}{{D_{a} E_{a} }} \cdot \tau_{m} \left( x \right) = 0$$

#### Relationship between shear stress and relative displacement of the anchorage section under the non-limit active state

To analyze the relationship between the shear stress and relative displacement of the anchorage section, we need to analyze the non-linear variation process between the value of the shear strength exertion and the relative displacement. According to the interaction mechanism between the anchorage section and surrounding soil, the value of the shear strength exertion on the surface of the anchorage section $\tau_{m}$ can be expressed as follows:

26 $$\tau_{m} = \sigma^{a} \tan \varphi_{m} + c_{m}$$

where $\tau_{m}$ is the value of the shear strength exertion under the non-limit active state; $\varphi_{m}$ is the value of the internal friction angle under the non-limit active state, which can be obtained from Eq. (14); $c_{m}$ is the value of the cohesion exertion under the non-limit active state, which can be obtained from Eq. (17); $\sigma^{a}$ is the normal compressive stress around anchor hole wall, and can be expressed as follows^24^:

27 $$\sigma^{a} = K^{a} \gamma h$$

where $K^{a}$ is the earth pressure coefficient of the hole wall in the anchorage section (generally between 0.5 and 1.0); $h$ is the thickness of the soil layer above the anchorage section; $\gamma$ is the soil weight of the anchorage section. Therefore, Eq. (25) can be expressed as follows:

28 $$\tau_{m} = K^{a} \gamma h\tan \varphi_{m} + c_{m}$$

According to Eqs. (17) and (28), it can be obtained as follows:

29 $$\tau_{m} = K^{a} \gamma h\tan \varphi_{m} + \frac{{\tan \varphi {}_{m}}}{{\tan \varphi_{f} }}c_{f}$$

Then, according to the variation process of the relative displacement of the anchorage section, the displacement specific gravity $\eta$ is defined as the ratio of relative displacement of the anchorage section, that is:

30 $$\eta = \frac{{S_{as} }}{{S_{as}^{f} }}$$

where $S_{as}$ is the relative displacement of anchorage section under the non-limit active state; $S_{as}^{f}$ is the relative displacement of anchorage section under limit active state, and can be expressed as follows:

31 $$S_{as}^{f} = \frac{{\pi D_{a} \tau_{f} }}{{G_{s} }}$$

where $\tau_{f}$ is the value of shear strength under the limit active state; $D_{a}$ is the diameter of anchorage section; $G_{s}$ is the shear modulus of the interface between anchorage section and surrounding soil, and can be obtained as follows:

32 $$G_{s} = \frac{{E_{s}^{a} }}{{2\left( {1 + \nu } \right)}}$$

where $E_{s}^{a}$ is the elastic modulus of soil; $\nu$ is the Poisson’s ratio of soil.

To establish the non-linear relationship between the shear strength and the relative displacement, Eq. (29) can be transformed into:

33 $$\tau_{m} = \frac{{\sin \varphi_{m} }}{{\sqrt {1 - \sin^{2} \varphi_{m} } }} \cdot \left( {K^{a} \gamma h + \frac{{c_{f} }}{{\tan \varphi_{f} }}} \right)$$

At the same time, in Eq. (13):

34 $$\left\{ \begin{gathered} A = \left( {1 - R_{f} } \right) \cdot \left( {1 - K_{0} } \right) \cdot \left( {1 + \sin \varphi_{f} } \right) \hfill \\ B = \left( {1 - K_{0} } \right) \cdot \left( {1 + \sin \varphi_{f} } \right) \cdot R_{f} + \left( {1 + K_{0} } \right) \cdot \sin \varphi_{f} - \left( {1 - K_{0} } \right) \hfill \\ C = \left( {1 - R_{f} } \right) \cdot \left( {1 + K_{0} } \right) \cdot \left( {1 + \sin \varphi_{f} } \right) \hfill \\ D = \left( {1 + K_{0} } \right) \cdot \left( {1 + \sin \varphi_{f} } \right) \cdot R_{f} - \left( {1 + K_{0} } \right) \cdot \sin \varphi_{f} + \left( {1 - K_{0} } \right) \hfill \\ \end{gathered} \right.$$

Therefore, Eqs. (14), (30), and (34) can be obtained as follows:

35 $$\sin \varphi_{m} = \frac{{A + \frac{B}{{S_{as}^{f} }} \cdot S_{as} }}{{C + \frac{D}{{S_{as}^{f} }} \cdot S_{as} }}$$

By substituting Eq. (35) into Eq. (33), it can be obtained as follows:

36 $$\tau_{m} = \left( {K^{a} \gamma h + \frac{{c_{f} }}{{\tan \varphi_{f} }}} \right) \cdot \frac{{A + \frac{B}{{S_{as}^{f} }} \cdot S_{as} }}{{\sqrt {\left( {C + \frac{D}{{S_{as}^{f} }} \cdot S_{as} } \right)^{2} - \left( {A + \frac{B}{{S_{as}^{f} }} \cdot S_{as} } \right)^{2} } }}$$

Equation (36) is the expression of a non-linear relationship between the shear strength of soil and the relative displacement of the anchorage section under the non-limit active state.

By substituting Eq. (36) into Eq. (25), it can be obtained as follows:

37 $$\frac{{d^{2} S_{as} \left( x \right)}}{{dx^{2} }} - \frac{4}{{DE_{a} }} \cdot \left( {K^{a} \gamma h + \frac{{c_{f} }}{{\tan \varphi_{f} }}} \right) \cdot \frac{{A + \frac{B}{{S_{as}^{f} }} \cdot S_{as} \left( x \right)}}{{\sqrt {\left( {C + \frac{D}{{S_{as}^{f} }} \cdot S_{as} \left( x \right)} \right)^{2} - \left( {A + \frac{B}{{S_{as}^{f} }} \cdot S_{as} \left( x \right)} \right)^{2} } }} = 0$$

Equation (37) is the second-order differential equation of relative displacement distribution along the length of the anchorage section. Therefore, according to the force characteristics of the anchorage section, the boundary condition of Eq. (37) can be obtained as follows:

38 $$\left\{ \begin{gathered} p\left( x \right)\left| {_{x = 0} } \right. = \left( {\frac{{\pi D_{a}^{2} E_{a} }}{4} \cdot \frac{{{\text{d}}S_{ae} \left( x \right)}}{{{\text{d}}x}}} \right)\left| {_{x = 0} = - P} \right. \hfill \\ p\left( x \right)\left| {_{{x = L_{a} }} } \right. = \left( {\frac{{\pi D_{a}^{2} E_{a} }}{4} \cdot \frac{{{\text{d}}S_{ae} \left( x \right)}}{{{\text{d}}x}}} \right)\left| {_{{x = L_{a} }} = 0} \right. \hfill \\ \end{gathered} \right.$$

By simplifying Eq. (38), the first derivative boundary value of relative displacement between the anchorage section and surrounding soil $S_{as}$ is obtained as follows:

39 $$\left\{ \begin{gathered} \left( {\frac{{{\text{d}}S_{ae} \left( x \right)}}{{{\text{d}}x}}} \right)\left| {_{x = 0} } \right. = \frac{ - 4P}{{\pi D_{a}^{2} E_{a} }} \hfill \\ \left( {\frac{{{\text{d}}S_{ae} \left( x \right)}}{{{\text{d}}x}}} \right)\left| {_{{x = L_{a} }} } \right. = 0 \hfill \\ \end{gathered} \right.$$

By using MATLAB^25,26^ (the code can be found in Appendix B), the boundary condition (Eq. (39)) is substituted into the second-order differential equation (Eq. (37)) of the relative displacement distribution along the length of the anchorage section to solve, and then the relative displacement size and distribution form of the anchor section considering the process of shear strength exertion can be obtained.

Based on the above analysis of the anchor’s force and displacement, the free section is mainly affected by the external tension, and the anchorage section balances the external tension transmitted by the free section through the shear stress between the anchorage section and the surrounding soil. Therefore, according to the anchor’s various forces, its total displacement is divided into the free section displacement and the anchorage section displacement, as shown in Eqs. (18), (20), and (21). The displacement of the free section is calculated according to the elastic theory, as shown in Eq. (19), premised on considering the process of shear strength exertion, the displacement calculation of the anchorage section is analyzed according to the non-linear theory, as shown in Eq. (37). Based on the above analysis of force and displacement, we can establish the force and displacement change of the anchor under the non-limit active state and identify the anchor’s actual working state in real time.

## Comparative analysis and discussion of examples

**Figure 4:**
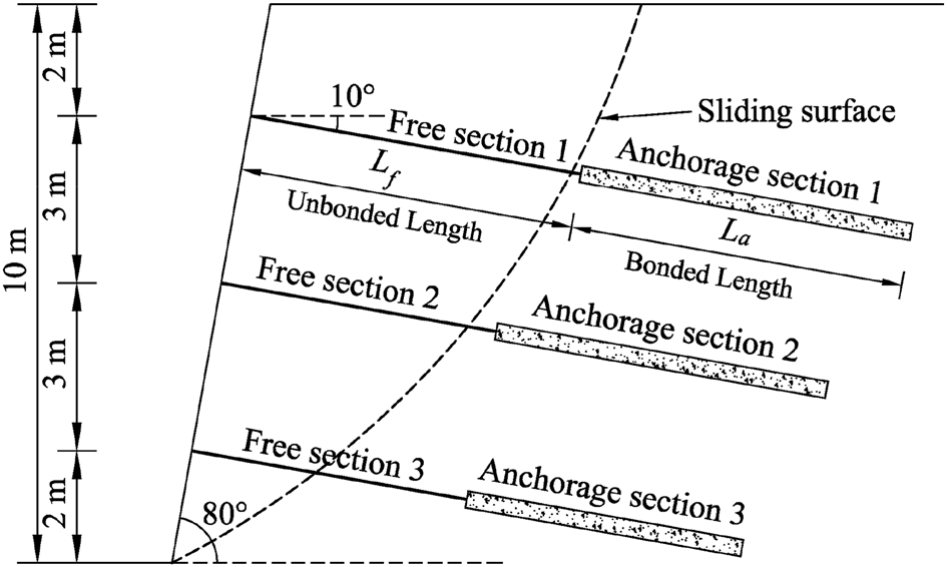
The diagram of reinforced slope.

To verify the rationality of the calculation method for the force and displacement of the anchor proposed in this paper, this section takes a homogeneous single-stage frame anchor reinforcement slope project as the analysis object (see Fig. 4) and uses the paper’s calculation method and the hyperbolic function model calculation method^8,12,15,16,27^ to compare the anchor’s force and displacement under various loads. Because the paper’s research object is the anchor, the influence of the frame structure is not considered in the analysis or discussion for this example.

### Selection of calculation parameters

The height of the slope is 10 m and its incline is 80°. There are 3 anchors at positions 2 m, 5 m, and 8 m on the side slope. The anchors’ inclination angles are all 10°. Table 1 shows the specific parameter of the anchor. In addition, the slope consists of uniformly distributed silty clay. Table 2 shows the corresponding soil parameters.

**Table 1:** Parameters of anchor.

Number	Diameter of steel bar	Anchor length	Unbonded length	Bonded length	Diameter of anchorage section
1	25 mm	12 m	6 m	6 m	150 mm
2	25 mm	11 m	5 m	6 m	150 mm
3	25 mm	10 m	5 m	5 m	150 mm

**Table 2:** Parameters of soil.

Soil	Weight $\gamma$	Cohesion $c_{f}$	Internal friction angle $\varphi_{f}$	Poisson's ratio $\nu$	Elastic modulus $E_{s}^{a}$
Silty Clay	16.4 kN/m^3^	18 kPa	25°	0.3	10.5 MPa

The body of the anchor is a steel bar and its elastic modulus $E_{s}$ is $2 \times 10^{5}$ MPa. The anchorage section is cement paste, and its elastic modulus $E_{g}$ is $2 \times 10^{4}$ MPa. According to Eq. (24), the effective elastic modulus of the anchorage section $E_{a}$ can be obtained as $2.5 \times 10^{4}$ MPa, in which the cross-sectional area of the anchor’s steel bar $A_{s}$ is $490.63$ mm^2^ and the cross-sectional area of the cement paste of the anchorage section $A_{g}$ is $17171.87$ mm^2^.

### Calculation method

The anchor’s force and displacement under various embedded depths and different loads are calculated and analyzed by using the calculation method in this paper. According to the above discussion about the composition of the anchor’s displacement, the calculation and analysis of the anchor’s force and displacement divide into the calculation and analysis of the force and displacement of the anchorage section and the calculation and analysis of the force and displacement of the free section.

#### Calculation and analysis of the force and displacement of the anchorage section

Considering the accuracy of the calculation and failure form of the anchor, the damage ratio $R_{f}$ is 0.95; the earth pressure coefficient of the hole in the wall in the anchorage section $K^{a}$ is 1.0. We need to solve other relevant calculation parameters according to the relevant formulas in this paper. Table 3 shows the calculation results.

**Table 3:** Calculation parameters.

Anchor	$\gamma$ kN/m^3^	$c_{f}$ kPa	$\varphi_{f}$ °	$R_{f}$	$K_{0}$	$D_{a}$ mm	$E_{a}$ MPa	$G_{s}$ MPa	$h$ m	$\tau_{f}$ kPa	$S_{as}^{f}$ mm	$A$	$B$	$C$	$D$
1	16.4	18	25	0.95	0.53	150	$2.5 \times 10^{4}$	4.04	3.5	44.77	5.2	0.03	0.81	0.11	1.89
2									6.5	67.71	7.9				
3									9.5	90.65	10.6				

By substituting the parameter values in Table 3 into Eq. (37), the second-order differential equation of the relative displacement distribution along the length of the anchorage section at different positions of the slope can be obtained. There is a direct relationship between the boundary conditions and the external tension of the anchor through the analysis Eq. (39). Therefore, to obtain the tension of the anchor, it is necessary to determine its ultimate bearing capacity. For this example, the anchor’s ultimate bearing capacity of the steel bar is 265 kN, and the pull-out resistance provided by the anchorage section is generally calculated according to Eq. (40). The specific calculation results are shown in Table 4.

40 $$F_{a} = \pi D\tau_{f} \cdot L_{a}$$

**Table 4:** General calculation of the anchor’s bearing capacity.

Anchor	Bearing capacity of anchor’s steel bar	Pullout resistance	Ultimate bearing capacity
1	265 kN	126.51 kN	126.51 kN
2	265 kN	191.34 kN	191.34 kN
3	265 kN	213.48 kN	213.48 kN

Because the relative displacement of the anchorage section does not change significantly when the anchor is subjected to a small external load, to accurately determine the relative displacement variation law of the anchorage section along the length of the anchorage section under the action of external load, the analysis is based on the calculated value of the ultimate bearing capacity of the anchor at various positions (see Table 4). We selected the external load close to the anchor’s ultimate bearing capacity (for a better and intuitive analysis of the variation law, the external load is selected from 70% of the ultimate load) to analyze the relative displacement and shear stress of the anchorage section (the value of shear strength exertion). Table 5 shows the specific external load value.

**Table 5:** External load on anchor bearing.

Anchor	External load (kN)											
	90	110	120	125	150	170	180	185	190	200	210	213
1	√	√	√	√	–	–	–	–	–	–	–	–
2	–	–	–	–	√	√	–	√	√	–	–	–
3	–	–	–	–	–	–	√	–	–	√	√	√

**Figure 5:**
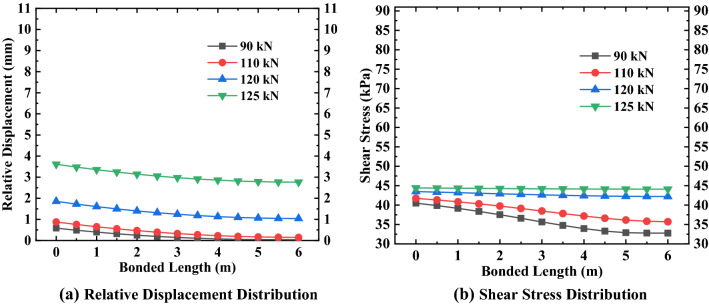
Relative displacement and shear stress distribution of the first anchor.

**Figure 6:**
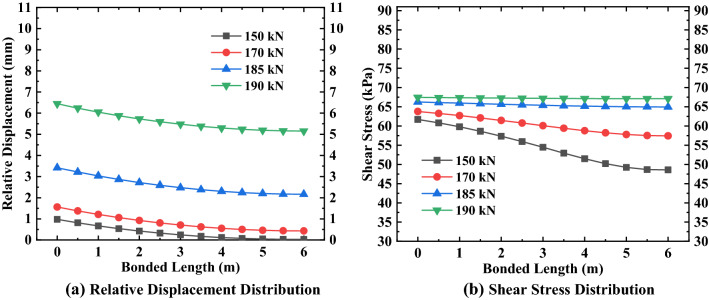
Relative displacement and shear stress distribution of the second anchor.

**Figure 7:**
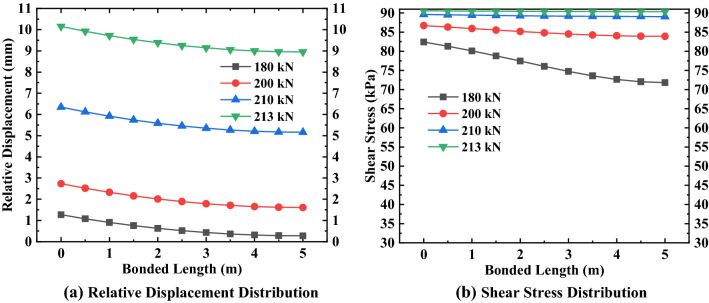
Relative displacement and shear stress distribution of the third anchor.

By substituting the relevant parameter values in Tables 1, 2, 3, 4, 5 into Eqs. (37) and (39), and using MATLAB to solve, the specific values and variation rules of relative displacement of the anchorage section at various positions under different loads can be obtained, as shown in Figs. 5, 6, 7.

It can be seen from Figs. 5, 6, 7 that the maximum relative displacement and maximum shear stress of the anchorage section at various positions appear at the junction of the anchorage section and the free section (i.e. the top of the anchorage section). In addition, the relative displacement and shear stress of the anchorage section at various positions decreases gradually from the top of the anchorage section to the end of the anchorage section along its length, and the relationship between them presents the characteristics of non-linear attenuation. By analyzing the change rate of relative displacement and shear stress, it can be found that the change rate of the near ultimate load (that is, more than 70% of the ultimate load) is sharp, while the change rate of the first 80% of the anchorage section length is mainly concentrated, and the change rate of the last 20% of the anchorage section is relatively flat. Also, it can be seen from the above figures that the difference in shear stress at both ends of the anchorage section at various positions shows a decreasing trend when the external load gradually increases to the ultimate load, the shear stress distribution of the anchorage section presents a uniform distribution trend along the length of the anchorage section, and the maximum shear stress value gradually approaches the ultimate shear strength value of the surrounding soil. As for the relative displacement difference between the two ends of the anchorage section, it shows an increasing change law as the external load gradually increases to the limit load, and the maximum relative displacement is close to the corresponding relative displacement $S_{as}^{f}$ when the shear strength reaches the ultimate value.

To better understand the variation of the relative displacement and shear stress of the anchorage section under various loads at different positions, the data in Figs. 5, 6, 7 are analyzed and sorted (see Tables 6, 7, 8).

**Table 6:** Variation of anchorage section of the first anchor.

Anchorage section		External load (kN)			
		90	110	120	125
Top 80% of bonded length	$\Delta S_{as}$(mm)	0.54	0.71	0.79	0.83
	$\Delta \tau_{m}$(kPa)	7.60	5.59	1.22	0.31
Last 20% of bonded length	$\Delta S_{as}$(mm)	0.01	0.02	0.02	0.02
	$\Delta \tau_{m}$(kPa)	0.12	0.44	0.05	0.01
Bonded length	$\Delta S_{as}$(mm)	0.55	0.73	0.81	0.85
	$\Delta \tau_{m}$(kPa)	7.72	6.03	1.27	0.32

**Table 7:** Variation of anchorage section of the second anchor.

Anchorage section		External load (kN)			
		150	170	185	190
Top 80% of bonded length	$\Delta S_{as}$(mm)	0.93	1.10	1.22	1.25
	$\Delta \tau_{m}$(kPa)	12.48	5.99	1.24	0.34
Last 20% of bonded length	$\Delta S_{as}$(mm)	0.02	0.03	0.03	0.04
	$\Delta \tau_{m}$(kPa)	0.64	0.37	0.05	0.01
Bonded length	$\Delta S_{as}$(mm)	0.95	1.13	1.25	1.29
	$\Delta \tau_{m}$(kPa)	13.12	6.36	1.29	0.35

**Table 8:** Variation of anchorage section of the third anchor.

Anchorage section		External load (kN)			
		180	200	210	213
Top 80% of bonded length	$\Delta S_{as}$(mm)	0.96	1.08	1.14	1.16
	$\Delta \tau_{m}$(kPa)	9.77	2.64	0.53	0.21
Last 20% of bonded length	$\Delta S_{as}$(mm)	0.04	0.04	0.05	0.05
	$\Delta \tau_{m}$(kPa)	0.82	0.16	0.03	0.01
Bonded length	$\Delta S_{as}$(mm)	1.00	1.12	1.19	1.21
	$\Delta \tau_{m}$(kPa)	10.59	2.80	0.56	0.22

By analyzing Tables 6, 7, 8, we found that the variation in the relative displacement of the anchorage section at various positions increases with an increase of external load, which indicates that the anchorage section provides sufficient shear stress to offset the external load by increasing the relative displacement. The variation of the shear stress of the anchorage section decreases with increasing load, which shows that the shear stress of the anchorage section is gradually distributed evenly along the length of the anchorage section under the action of the external load and relative displacement, to offset the external load. For anchors located in different positions, the variation of the relative displacement and shear stress of the anchorage section increases with the increase in the embedded depth of the anchor under the same load-limit load ratio, which indicates that the embedded depth of the anchor has a direct influence on the variation range of the relative displacement and shear stress of the anchorage section.

After analyzing the variations in the relative displacement and shear stress in several areas of the anchorage section, we found that the relative displacement variation of the first 80% of the anchorage section accounts for more than 96% of total relative displacement variation; at the same time, the shear stress variation of the first 80% of the anchorage section accounts for more than 92% of the total shear stress variation. This shows that the main variation range of relative displacement and shear stress exists in the first 80% of the anchorage section, while the variation of the remaining 20% of the anchorage section is small.

To show the influence of the anchorage section on the displacement and force of the overall anchor under different loads more intuitively, this paper selected the junction between the anchorage section and the free section (that is, the top of the anchorage section) as its research object, and analyzed the displacement and force of the area under various loads.

**Figure 8:**
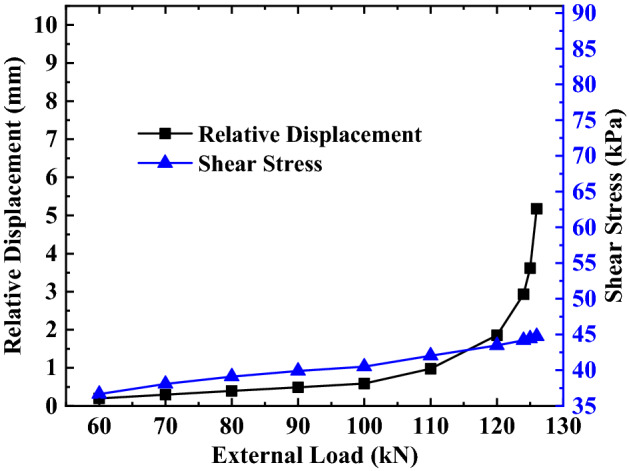
Analysis of the top of the anchorage section of the first anchor under different loads.

**Figure 9:**
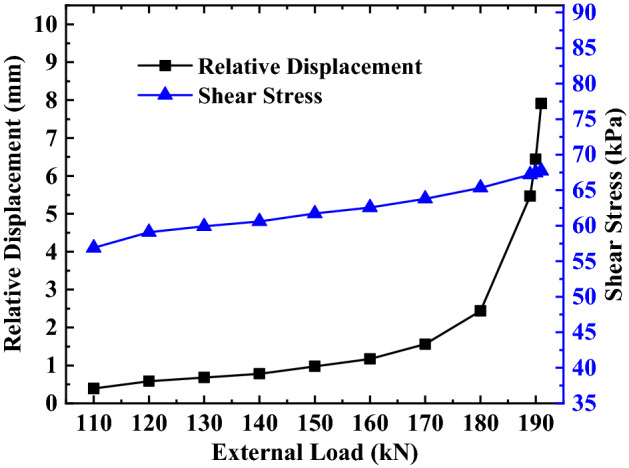
Analysis of the top of the anchorage section of the second anchor under different loads.

**Figure 10:**
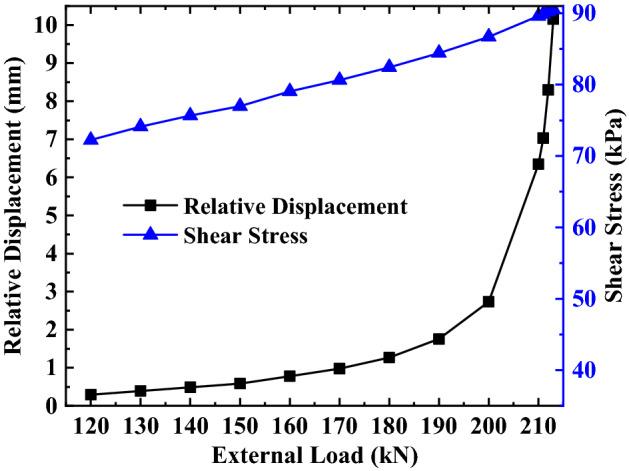
Analysis of the top of the anchorage section of the third anchor under different loads.

It can be seen from Eq. (21) that the total displacement of the anchorage section $S_{a}$ at the junction of the anchorage section and the free section is equal to the relative displacement $S_{as}$ between the top of the anchorage section and the surrounding soil, and the side friction resistance at this position against the external load is provided by the shear stress between the anchorage section and the surrounding soil. Therefore, the analysis of the displacement and force at the junction of the anchorage section and the free section under various loads is essentially the analysis of the relative displacement and shear stress at this position. To summarize, according to this paper’s calculation method, the parameter values of this example are substituted into Eqs. (37) and (39) and solved using MATLAB, and the relative displacement and shear stress calculation values at the junction between the anchorage section and the free section under various loads can be obtained. The variation curve is drawn according to the calculation values, as shown in Figs. 8, 9, 10.

It can be seen from Figs. 8, 9, 10 that the relative displacement and shear stress at the top of the anchorage section increases non-linearly with the external load’s increase, but the rate of variation between them and the external load is not the same.

For the shear stress at the top of the anchorage section, the rate of variation shows no significant change in the process of increasing the external load, and the trend of the overall variation is more uniform. The above changes indicate that different loads have a greater impact on the shear stress.

For the relative displacement at the top of the anchorage section, when the external load is less than 90% of the ultimate load, the relative displacement in this area changes slightly with any increase in the external load. However, when the external load is more than 90% of the ultimate load, the relative displacement in this area changes sharply with the increase in the external load. The above changes indicate that load has less influence on relative displacement when the external load is less than 90% of the ultimate load, while the load has a greater influence on the relative displacement when the external load is more than 90% of the ultimate load.

By comparing and analyzing the variation of relative displacement and shear stress at the top of the anchorage section with an external load at different depths, it can be found that (1) the variation range of relative displacement and shear stress at the top of the anchorage section increases with a continuous increase of the anchor’s embedded depth; (2) Under the same load-limit load ratio, the variation trend of relative displacement and shear stress at the top anchorage section remains unchanged with an increase in the anchor’s embedded depth of the anchor; (3) Under the same embedded depth, the variation trend of relative displacement and shear stress at the top of anchorage section is substantially different; that is, the relative displacement has changed with the increase of the external load, but the shear stress is an approximately linear increase with the increase of external load.

**Figure 11:**
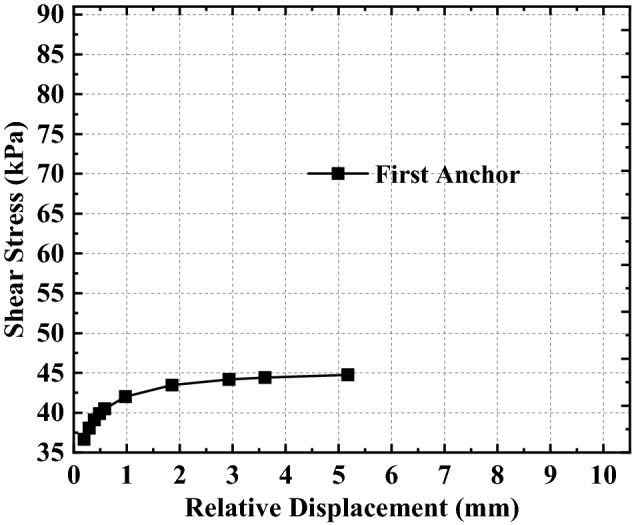
Analysis of the top of the anchorage section of the first anchor.

**Figure 12:**
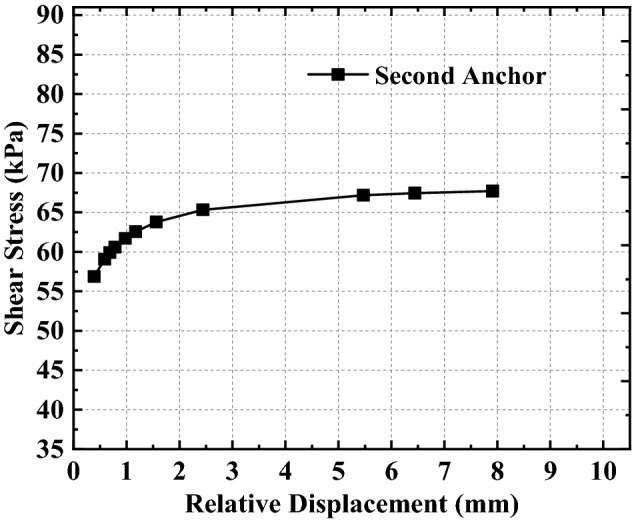
Analysis of the top of the anchorage section of the second anchor.

**Figure 13:**
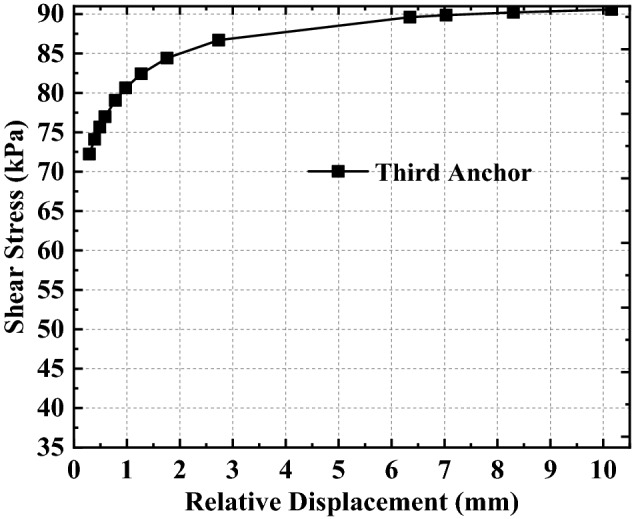
Analysis of the top of the anchorage section of the third anchor.

Based on the above analysis, it can be found that the relationship between shear stress and relative displacement is not linear as assumed in previous studies, but a complex non-linear relationship (as shown in Eq. (39)). To better analyze the non-linear relationship between relative displacement and shear stress expressed by Eq. (39), the relative displacement and shear stress curves at the top of the anchorage section with different embedded depths are drawn by combining the relative displacement and shear stress data in Figs. 7, 8, 9 (see also Figs. 11, 12, 13).

From Figs. 11, 12, 13, combined with Eq. (39), the variation of shear stress with relative displacement at the junction of the free section and anchorage section (i.e., the top of the anchorage section) can be summarized as follows:

1. The shear stress increases non-linearly with the increase in relative displacement; that is, the shear stress first increases rapidly with relative displacement increase, then gradually.
2. When the relative displacement is within 60% of the maximum relative displacement, the shear stress changes significantly with the relative displacement increase (the amount of shear stress variation accounts for more than 98% of the total shear stress variation).
3. When the relative displacement is more than 60% of the maximum relative displacement, the shear stress changes only slightly with the relative displacement increase (the amount of shear stress variation accounts for less than 2% of the total shear stress variation);
4. The variation rule of shear stress-relative displacement does not change with the anchor’s different embedded depths, but it has a greater influence on the shear stress variation range; that is, the difference in shear stress variation interval increases with the increase in the anchor’s embedded depth.

Based on the above analysis of the relative displacement and shear stress variation trend of the anchorage section under different embedded depths, the anchorage section’s location and loads, the following non-linear variation laws of shear stress and relative displacement of the anchorage section can be obtained:

1. The maximum relative displacement and maximum shear stress of the anchorage section occur at the junction of the anchorage section and free section (the top of the anchorage section), and the relative displacement and shear stress of the anchorage section gradually decreases from the top of the anchorage section to its end along the length of anchorage section. The changing relationship shows non-linear attenuation characteristics.
2. The relative displacement variation of the anchorage section increases with the continuous increase in the external load, while the shear stress variation of the anchorage section decreases with the load increase.
3. Under the same load-to-limit ratio, the relative displacement and shear stress of the anchorage section increases with the increase of the anchor’s embedded depth.
4. The main variation range of relative displacement and shear stress of the anchorage section exists in the first 80% of the anchorage section, and the second 20% of the anchorage section is uniformly distributed.
5. Both the relative displacement and shear stress at the top of the anchorage section increases non-linearly with an increase in the external load. The shear stress changes more uniformly, while the relative displacement has an obvious mutation point. At the same time, the variation range of the relative displacement and shear stress at the top of the anchorage section increases with an increase in the embedded depth of the anchor.
6. With the increase of the relative displacement, the shear stress shows a non-linear change trend that first increases rapidly and then gradually, and the rapid increase area is the first 60% of the maximum relative displacement.

According to the above variation law, the force distribution and displacement variation range of the anchorage section under different loads can be determined. At the same time, according to the correlation analysis of Eq. (21), the displacement at the junction of the anchorage section and the free section (the top of the anchorage section) is selected as the displacement component value of the anchorage section to the whole anchor displacement. Finally, combined with the displacement of the free section, the displacement variation of the whole anchor under different loads can be obtained.

#### Calculation and analysis of the force and displacement of free section

According to the calculation and analysis of the force and displacement of the free section in **Sect. 3.1**, their characteristics can be obtained as follows: the free section is directly subject to the external load, which is transferred to the anchorage section. The free section is elastically deformed during the transfer-the anchor’s body does not reach the ultimate bearing capacity. Therefore, it is considered that the displacement value of the free section is its elastic deformation value when the external load does not reach the ultimate bearing capacity of the anchor’s body. Finally, according to Eq. (19) and the relevant parameter values in this example, the displacement value of the free section $S_{fe}$ can be obtained, as shown in Table 9, where $L_{f}$ is 6 m (the first anchor) or 5 m (the second and third anchors); $E_{s}$ is $2 \times 10^{5}$ MPa; $A_{s}$ is $490.63$ mm^2^.

**Table 9:** The anchor’s free section displacement value.

$S_{fe}$(mm)	External load (kN)																							
	60	70	80	90	100	110	120	124	125	126	130	140	150	160	170	180	189	190	191	200	210	211	212	213
1	3.67	4.28	4.89	5.50	6.11	6.73	7.34	7.58	7.64	7.70	–	–	–	–	–	–		–	–	–	–	–	–	–
2	–	–	–	–	–	5.61	6.11	–	–	–	6.62	7.13	7.64	8.15	8.66	9.17	9.63	9.68	9.73	–	–	–	–	–
3	–	–	–	–	–	–	6.11	–	–	–	6.62	7.13	7.64	8.15	8.66	9.17	–	9.68	–	10.19	10.70	10.75	10.80	10.85

#### Calculation and analysis of the anchor’s force and displacement

It can be seen from **Sect. 3** and Eqs. (18), (20), (21) that the displacement of the anchor $S^{a}$ is composed of the displacement of the free section $S_{fe}$ and the displacement of the anchorage section $S_{a}$, in which the displacement of the latter $S_{a}$ is equal to the relative displacement $S_{as}$ between the top of the anchorage section and the surrounding soil. Therefore, in this example, the displacement of the anchor under different loads can be calculated according to Sect. 4.2.1 and Sect. 4.2.2. The results are shown in Tables 10, 11, 12.

**Table 10:** Displacement of the first anchor.

Displacement (mm)		External load (kN)									
		60	70	80	90	100	110	120	124	125	126
Free section	$S_{fe}$	3.67	4.28	4.89	5.50	6.11	6.73	7.34	7.58	7.64	7.70
	Ratio	94.95%	93.59%	92.61%	91.85%	91.26%	87.32%	79.82%	72.13%	67.90%	59.82%
Anchorage section	$S_{as} \left| {_{x = 0} } \right.$	0.20	0.29	0.39	0.49	0.59	0.98	1.86	2.93	3.61	5.18
	Ratio	5.05%	6.41%	7.39%	8.15%	8.74%	12.68%	20.18	27.87%	32.10%	40.18%
Total of anchor		3.86	4.57	5.28	5.99	6.70	7.70	9.19	10.51	11.26	12.88

**Table 11:** Displacement of the second anchor.

Displacement (mm)		External load (kN)										
		110	120	130	140	150	160	170	180	189	190	191
Free section	$S_{fe}$	5.61	6.11	6.62	7.13	7.64	8.15	8.66	9.17	9.63	9.68	9.73
	Ratio	93.48%	91.26%	90.65%	90.13%	88.67%	87.43%	84.72%	78.98%	63.78%	60.03%	55.16%
Anchorage section	$S_{as} \left| {_{x = 0} } \right.$	0.39	0.59	0.68	0.78	0.98	1.17	1.56	2.44	5.47	6.45	7.91
	Ratio	6.52%	8.74%	9.35%	9.87%	11.33%	12.57%	15.28%	21.02%	36.22%	39.97%	44.84%
Total of anchor		6.00	6.70	7.31	7.91	8.62	9.32	10.22	11.61	15.10	16.13	17.64

**Table 12:** Displacement of the third anchor.

Displacement (mm)		External load (kN)												
		120	130	140	150	160	170	180	190	200	210	211	212	213
Free section	$S_{fe}$	6.11	6.62	7.13	7.64	8.15	8.66	9.17	9.68	10.19	10.70	10.75	10.80	10.85
	Ratio	95.43%	94.43%	93.59%	92.88%	91.26%	89.87%	87.84%	84.63%	78.84%	62.77%	60.46%	56.55%	51.66%
Anchorage section	$S_{as} \left| {_{x = 0} } \right.$	0.29	0.39	0.49	0.59	0.78	0.98	1.27	1.76	2.73	6.35	7.03	8.30	10.16
	Ratio	4.57%	5.57%	6.41%	7.12%	8.74%	10.13%	12.16%	15.37%	21.16%	37.23%	39.54%	43.45%	48.34%
Total of anchor		6.41	7.01	7.62	8.23	8.93	9.64	10.44	11.44	12.93	17.05	17.78	19.10	21.01

According to the composition and variation of the anchor’s displacement under different loads in Tables 10, 11, 12, it can be found that the anchor’s displacement under different embedded depths has the same variation law as follows:

1. With an increasing load, the overall displacement of the anchor presents a non-linear variation trend of an approximate uniform increase followed by an abrupt increase.
2. According to the variation law of the free section’s displacement and the anchorage section’s displacement with load, it can be found that the variation trend of the anchor’s displacement is mainly affected by the variation trend of the anchorage section’s displacement. This variation is almost the same as the variation of relative displacement at the top of the anchored section—all showing a non-linear increase trend.

At the same time, according to the variation in the proportion of the displacement of the anchorage section and the displacement of the free section in the above Tables 10, 11, 12, the following rules can be determined:

1. The proportion of each component in the displacement of the anchor changes with the load increase, but in general, the displacement of the free section is the principal part of the anchor’s displacement, and its proportion is more than 50%.
2. The displacement of the free section increases linearly with the load increase, but its proportion decreases non-linearly.
3. As for the displacement of the anchor section, the proportion of the displacement of the anchor section increases non-linearly with the increase of the external load, and its proportion can increase to between 40 and 48% when the external load is the anchor’s ultimate pulling force, which constitutes an important part of the anchor’s displacement.

The calculation of the anchor’s force and displacement consists of two parts: the calculation and analysis of the force and displacement of the free section and anchorage section.

For the calculation of the former, because the anchor’s body is the steel bar and the free section is the anchor’s body, it is considered that the free section is elastically deformed when the ultimate bearing capacity of the anchor is not reached. According to Eq. (19), the calculated values of stress and displacement of the free section can be obtained, which is relatively simple.

For the calculation of the stress and displacement of the anchor section, because the force here is more complex and the shear stress and relative displacement of the anchor section have a non-linear relationship (as shown in Eq. (36)), the calculation of the force and displacement of the anchor section is more complex and a key part of the anchor’s force and displacement calculation. Therefore, the non-linear relationship between the shear stress and relative displacement of anchorage section is discussed in detail with specific calculation examples. The calculated values and variation trends of the relative displacement and shear stress of the anchor section under different embedment depths, different anchorage section locations, and different loads are obtained. Thus, the anchor’s force and displacement are more accurately calculated and analyzed.

### Calculation method of the hyperbolic model

To verify the rationality of the calculation method in this paper, the hyperbolic function model commonly used in the relevant literature^8,12,15,16,27^ is selected to calculate and analyze the anchor’s force and displacement. According to the existing literature, most studies^8,16^ assume that the relationship between shear stress and relative displacement is a linear elastic distribution; that is,

41 $$q = \pi D_{a} \tau = G_{s} S_{as}$$

The force analysis of the anchorage section is carried out, and the calculation formulas of relative displacement and shear stress are obtained; that is,

42 $$\left\{ \begin{gathered} S_{as} = \frac{4P}{{\pi D_{a} E_{a} \cdot \sqrt {\frac{{4G_{s} }}{{\pi E_{a} }}} }} \cdot \frac{{\cosh \left( {\sqrt {\frac{{4G_{s} }}{{\pi E_{a} }}} \cdot \frac{{L_{a} - x}}{{D_{a} }}} \right)}}{{\sinh \left( {\sqrt {\frac{{4G_{s} }}{{\pi E_{a} }}} \cdot \frac{{L_{a} }}{{D_{a} }}} \right)}} \hfill \\ \tau_{m} = \frac{{\sqrt {\frac{{4G_{s} }}{{\pi E_{a} }}} \cdot P}}{{\pi D_{a}^{2} }} \cdot \frac{{\cosh \left( {\sqrt {\frac{{4G_{s} }}{{\pi E_{a} }}} \cdot \frac{{L_{a} - x}}{{D_{a} }}} \right)}}{{\sinh \left( {\sqrt {\frac{{4G_{s} }}{{\pi E_{a} }}} \cdot \frac{{L_{a} }}{{D_{a} }}} \right)}} \hfill \\ \end{gathered} \right.$$

where $x$ is the length of any point of anchorage section.

The analysis from Eq. (42) shows that the embedded depth of the anchor is not considered in the calculation of the shear stress and relative displacement of the anchorage section. Therefore, to verify the rationality of this method, this paper uses only the hyperbolic function model to compare and analyze the second row of the anchor.

#### Comparative analysis of relative displacement and shear stress

**Figure 14:**
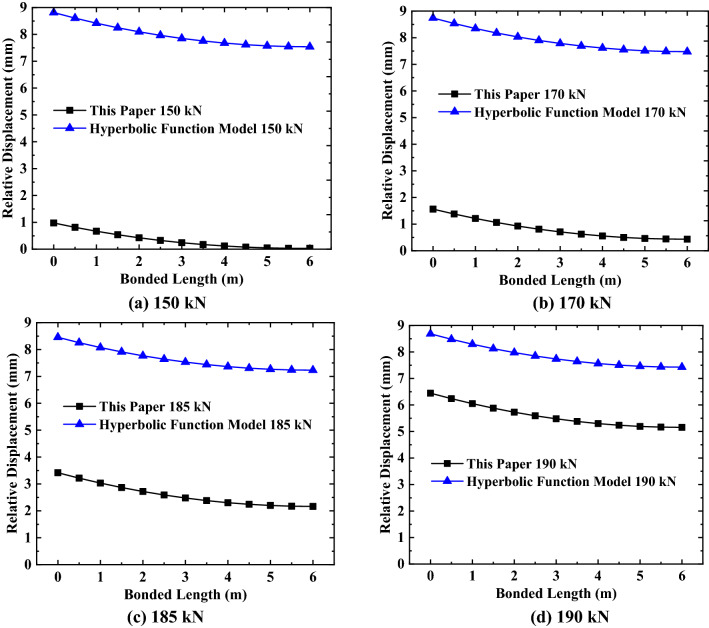
Comparison of relative displacement.

**Figure 15:**
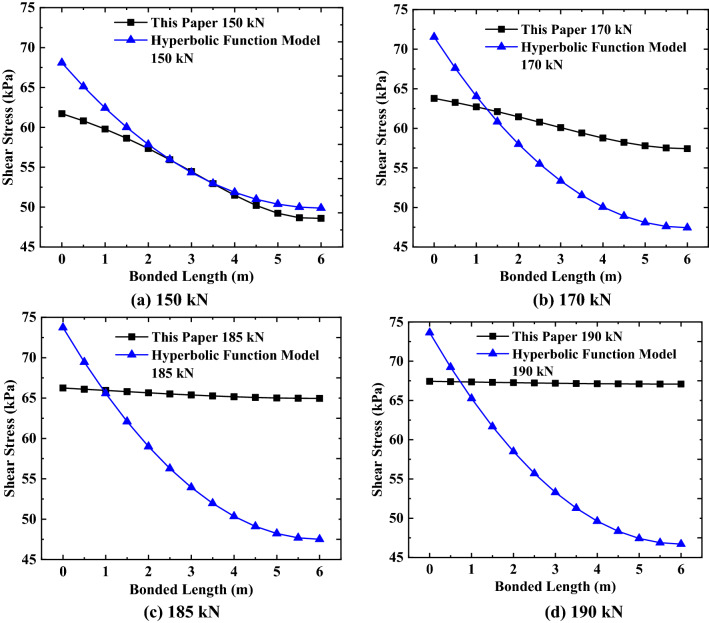
Comparison of shear stress.

By substituting the relevant parameter values of this example into Eqs. (41) and (42), the distribution law of shear stress and relative displacement of the anchorage section calculated by the hyperbolic function model is obtained, and compared with the variation law of shear stress and relative displacement of the anchorage section obtained by this calculation method, as shown in Figs. 14 and 15.

It can be seen from Fig. 14 that the variation trend of the relative displacement along the length of the anchorage section of the paper’s calculation method is the same as that calculated by the hyperbolic function model. This shows the variation law gradually decreasing with the increase of the length of the anchorage section, and the variation rate of the two calculation methods along the length of the anchorage section is similar. By analyzing the relative displacement values of the same position of the anchorage section calculated by this paper’s method and the hyperbolic function model, it is found that the relative displacement values calculated by the two methods differ, but the difference of the relative displacement decreases with an increase in the external load. When the external load is 185 kN, the maximum relative displacement of the hyperbolic function model exceeds the limit value of the theoretical calculation (7.9 mm), while in the relative displacement of this method before reaching the limit load is less than the limit value of the relative displacement, which is closer to the actual relative displacement variation of the anchorage section.

It can be seen from Fig. 15 that when the shear stress calculated by the hyperbolic function model does not reach the limit value of the soil shear strength (67.71 kPa), the variation law of the shear stress calculated by the method in this paper is the same as the variation law of the shear stress calculated by the hyperbolic function model (as shown in Fig. 15a, b). This shows the variation law of non-linear reduction along the length of the anchorage section, and the variation trend is similar. When the shear stress calculated by the hyperbolic function model exceeds the limit value of the soil shear strength (at this time, the external load does not reach the limit value that the anchorage section can bear), the shear stress calculated by the hyperbolic function model keeps the variation law in Fig. 15a, b (also shown in Fig. 15c, d), and there is no obvious mutation near the limit value of the soil shear strength. By comparing the shear stress distribution values obtained by this paper’s calculation method, it can be found that as the external load approaches the ultimate load of the anchorage section, the shear stress distribution gradually changes from a non-linear decrease to a uniform distribution along the anchorage section’s length, and the maximum shear stress also approaches the ultimate value of the soil shear strength. This change in the distribution trend is more consistent with the shear stress distribution of the anchorage section.

This paper summarizes and analyzes the reasons of the difference between the relative displacement and shear stress of the anchorage section in the two methods:

1. In this paper, the anchor’s embedded depth is considered in the calculation of the relative displacement and shear stress of the anchorage section, but the hyperbolic function model does not consider the anchor’s embedded depth;
2. In this paper, the non-linear relationship between the relative displacement and shear stress is considered, but the hyperbolic function model assumes that the relationship between them is linear;
3. The paper’s calculation method derives the relationship between displacement and shear stress theoretically, but the hyperbolic function model uses assumptions to determine their relationship.

Therefore, compared with the hyperbolic function model calculation method, this paper’s calculation method is closer to the actual relative displacement and shear stress distribution form of the anchoring section and meets the theoretical limit value.

#### Comparative analysis of the anchor’s total displacement

**Figure 16:**
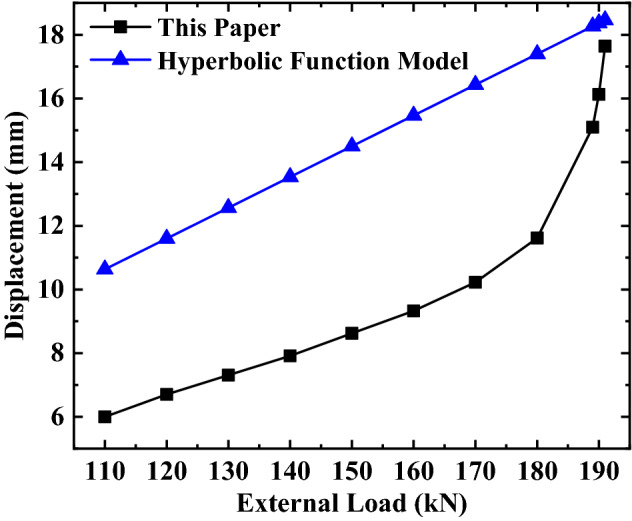
Comparison of the anchor’s total displacement.

The hyperbolic function model calculates the displacement of the free section in the same way as this paper, so the displacement value of the free section of the second row of the anchor in **Sect. 4.2.2** is used as the displacement value of the free section when calculating the anchor displacement. At the same time, the relative displacement at the top of the anchorage section is used as the displacement of the anchorage section in both methods. To summarize, the displacement values of the free section and the anchorage section are sorted, and the total displacement of the anchor in the two methods are obtained respectively, as shown in Fig. 16.

It can be seen from Fig. 16 that the anchor’s total displacement, obtained from the two calculation methods, increases when the external load increases, but the variation trend of the two methods is not the same. The anchor’s total displacement, calculated by the hyperbolic function model, shows a linear increase trend with the load’s increase. The anchor’s total displacement, calculated by this paper’s calculation method, shows a non-linear increasing trend with the load increase; that is, the increasing rate of the anchor’s total displacement is relatively gentle before reaching the limit load of 90%. What is more, the increasing rate of the anchor’s total displacement changes abruptly when the limit load is more than 90%. Also, under the same load, the anchor’s total displacement, calculated by the hyperbolic function model, is greater than the anchor’s total displacement calculated by this paper’s calculation method. However, on the whole, the difference of the anchor’s total displacement between the two methods decreases gradually with the load’s gradual increase. Approaching 90% of the ultimate load, the total displacement difference between the two methods is sharply reduced, until the external load reaches the ultimate load. The anchor’s total displacement obtained by the two calculation methods is similar.

## Conclusion

Based on the elastic theory for calculating the displacement of the free section, this paper proposes an analytical method to calculate the relative displacement and shear stress of the anchorage section by considering the process of soil shear strength exertion. A non-linear numerical relationship between shear stress and relative displacement of the anchorage section under the non-limit active state is also derived. Using the above theoretical analysis, this paper calculates the value and change trend of the shear stress and relative displacement of the anchorage section at various embedded depths, anchorage section positions, and loads. It improves the calculation method of the anchor’s displacement and force by considering the process of soil shear strength exertion under the non-limit active state. The main conclusions are as follows:

1. For the relative displacement of the anchorage section, when the external load does not reach the ultimate load, the relative displacement of the anchorage section obtained by the calculation method is less than the theoretical calculation value of the ultimate relative displacement. This reveals a variation law that decreases gradually with an increasing length of the anchorage section. However, the relative displacement of the anchorage section obtained by the hyperbolic function model exceeds the theoretical value of the ultimate relative displacement when the external load does not reach the ultimate load.
2. For the anchorage section’s shear stress (i.e. the value of the shear strength exertion of the soil around the anchorage section), when the external load does not reach the ultimate load, the calculated value obtained by this paper’s calculation method is less than the theoretically calculated value of the limit value of the soil shear strength, which shows the variation law of a non-linear decrease along the length of the anchorage section. As the external load continues to approach the ultimate load of the anchor, the shear stress distribution gradually changes from a non-linear reduction to a uniform distribution along the length of the anchorage section. However, for the shear stress of the anchorage section obtained by the hyperbolic function model, it exceeds the soil’s limit value of the shear strength when the external load has not reached the ultimate load, and the distribution form of the shear stress does not change significantly when the external load approaches the ultimate load of the anchor.
3. The anchor’s total displacement obtained by the calculation method shows a non-linear variation law, increasing slowly at first and then sharply with a continuous increase in the external load. However, the total displacement of the anchor calculated by the hyperbolic function model shows a linear increase with the external load.
4. Compared with the calculation method of the hyperbolic function model, the calculation method in this paper has the following advantages: a) The non-linear relationship between the shear stress of the anchorage section and the relative displacement of the anchorage section is deduced at the theoretical level by the paper’s calculation method; b) in the paper’s calculation method, the embedded depth of the anchor is added to the calculation formula of the shear stress and relative displacement; c) the calculated values and variation laws of the anchor’s total displacement, relative displacement and shear stress using the paper’s calculation method are more in line with the actual variation trend of the anchor and meet the theoretical limit value.

## Supplementary Information

Supplementary Information 1.

Supplementary Information 2.
