# Supplementary material for: Analysis of force and displacement of anchor systems under the non-limit active state
Source: Sci Rep. 2022 Jan 25;12:1306. doi: 10.1038/s41598-021-04668-9 (PMC8789931; doi:10.1038/s41598-021-04668-9)
Supplement: Supplementary file 1 — Supplementary Information 1. [file 41598_2021_4668_MOESM1_ESM.docx]

Detailed derivation of Eq. (25).

It can be obtained from Eq. (20):

$$S_{ae}=S_{a}-S_{as}$$

Find the second derivative of Eq. (A1):

$$\frac{\text{d}^{2}S_{as}\left(x\right)}{\text{d}x^{2}}=-\frac{\text{d}^{2}S_{ae}\left(x\right)}{\text{d}x^{2}}$$

Find the second derivative of Eq. (23):

$$\frac{\text{d}p\left(x\right)}{\text{d}x}=\frac{\pi D_{a}^{2}E_{a}}{4}\cdot\frac{\text{d}^{2}S_{ae}\left(x\right)}{\text{d}x^{2}}$$

Substitute Eq. (A3) into Eq. (22):

$$\frac{\text{d}^{2}S_{ae}\left(x\right)}{\text{d}x^{2}}=-\frac{4\tau_{m}\left(x\right)}{D_{a}E_{a}}$$

Eq. (25) can be obtained by substituting Eq. (A4) into Eq. (A2).
