# Supplementary material for: Analysis of force and displacement of anchor systems under the non-limit active state
Source: Sci Rep. 2022 Jan 25;12:1306. doi: 10.1038/s41598-021-04668-9 (PMC8789931; doi:10.1038/s41598-021-04668-9)
Supplement: Supplementary file 2 — Supplementary Information 2. [file 41598_2021_4668_MOESM2_ESM.docx]

This code consists of three parts, namely dynam.m, loss.m and main.m. In this code, x means length of anchorage section and y means $S_{as}\left(x\right)$. The specific MATLAB code is as follows:

dynam.m

function dy=dynam(x,y,para)

O=1.024e-4; % This value is calculated according to .

M=0.0336; % The value is calculated according to the calculation formula of $A$ in Eq. (34).

N=156.0769; % This value is calculated according to$B/S_{as}^{f}$, where $B$ is calculated according to Eq. (34).

P=0.1086; % The value is calculated according to the calculation formula of $C$ in Eq. (34).

Q=363.7308; % This value is calculated according to $D/S_{as}^{f}$, where D is calculated according to Eq. (34).

dy=zeros(2,1);

dy(1)=y(2);

numerator=O*(M+N*y(1));

denominator=(P+Q*y(1))^2-(M+N*y(1))^2;

denominator=sqrt(denominator);

dy(2)=numerator/denominator;

dy;

end

loss.m

function dy=loss(para)

xspan=0:0.0001:6; % The length of anchorage section

ini=[para(1),-0.0002491]; % The boundary condition calculation value of Eq. (37) is calculated by Eq. (38).

[x,y]=ode45(@dynam,xspan,ini);

len=length(x);

dy=y(len,2)^2;

dy=sqrt(dy);

end

main.m

clear all;clc;

xspan=0:0.0001:6;

para=2;

para=fminsearch(@loss,para)

ini=[para,-0.0002491];

[x,y]=ode45(@dynam,xspan,ini);

figure(1)

hold on;

plot(x,y(:,2))

xlabel('x');

ylabel('dy/dx');

ylim([-0.0002491,0.0001])

loss(para)

y1=y(:,1);

figure(2);

plot(x,y1);

xlabel('x');

ylabel('y');

model=polyfit(x,y(:,1),4);
